# Single-cell RNA sequencing reveals the communications between tumor microenvironment components and tumor metastasis in osteosarcoma

**DOI:** 10.3389/fimmu.2024.1445555

**Published:** 2024-09-11

**Authors:** Jiatong Li, Yang Bai, He Zhang, Ting Chen, Guanning Shang

**Affiliations:** ^1^ Department of Orthopedics, Shengjing Hospital of China Medical University, Shenyang, China; ^2^ Department of Nursing, Shengjing Hospital of China Medical University, Shenyang, China

**Keywords:** osteosarcoma, immunotherapy, therapy, RNA sequencing, TME

## Abstract

**Introduction:**

Osteosarcoma is a common type of bone cancer characterized by a poor prognosis due to its metastatic nature. The tumor microenvironment (TME) plays a critical role in tumor metastasis and therapy response. Therefore, our study aims to explore the metastatic mechanism of osteosarcoma, potentially opening new avenues for cancer treatment.

**Methods:**

In this study, we collected data from the GSE152048, GSE14359, and GSE49003 datasets. Differentially expressed genes (DEGs) were identified in osteosarcoma cases with primary and metastatic features using R software and the limma package. Gene ontology (GO) and Kyoto Encyclopedia of Genes and Genomes (KEGG) pathway enrichment analyses were performed to investigate metastasis-related genes. A protein–protein interaction (PPI) network was established using the STRING database to further analyze these metastasis-associated genes. The abundances of different cell types with a mixed cell population were estimated using the CIBERSORT approach. The scRNA-seq data were analyzed by the Seurat package in R software, and intercellular communications were elucidated using the CellChat R package.

**Results:**

In this study, 92 DEGs related to metastasis were identified, including 41 upregulated and 51 downregulated genes in both the GSE14359 and GSE49003 datasets. Metastasis-associated pathways were identified, including those involving the cyclin-dependent protein kinase holoenzyme complex, transferase complex, transferring phosphorus-containing groups, SCF ubiquitin ligase complex, and the serine/threonine protein kinase complex. KEGG and PPI network analyses revealed 15 hub genes, including Skp2, KIF20A, CCNF, TROAP, PHB, CKS1B, MCM3, CCNA2, TRIP13, CENPM, Hsp90AB1, JUN, CKS2, TK1, and KIF4A. Skp2 has been known as an E3 ubiquitin ligase involved in osteosarcoma progression. The proportion of CD8+ T cells was found to be higher in metastatic osteosarcoma tissues, and high expression of PHB was associated with a favorable prognosis in osteosarcoma patients. Additionally, 23 cell clusters were classified into eight cell types, including chondrocytes, MSC, T cells, monocytes, tissue stem cells, neurons, endothelial cells, and macrophages. The 15 hub genes were expressed across various cell types, and interactions between different cell types were observed.

**Conclusion:**

Our study reveals the intricate communication between tumor microenvironment components and tumor metastasis in osteosarcoma.

## 1 Introduction

Osteosarcoma is a common type of bone cancer that often occurs in the long bones of the legs and arms and is more prevalent among children and young adults. Symptoms of osteosarcoma include pain, swelling, and a noticeable lump in the affected bones (1). Treatment options include surgical excision, chemotherapy, radiation therapy, and targeted therapy (2, 3). Early detection and intervention are crucial for achieving better outcomes, which makes exploring the underlying mechanism of osteosarcoma vital (4). Although the exact cause of osteosarcoma is not fully understood, certain genetic and epigenetic factors may increase the risk of its development (5–7). It is essential to uncover the mechanisms of osteosarcoma development and reoccurrence in order to develop effective therapy for patients (8, 9).

Tumor metastasis is a key factor influencing therapeutic outcomes and prognosis in cancer patients (10, 11). Osteosarcoma is particularly characterized by its highly metastatic feature. Due to this tendency for metastasis, patients with osteosarcoma often experience poor treatment responses and worse prognosis. The 5-year survival rate for osteosarcoma is about 70%, but it drops to 20%–30% for patients with metastatic disease (12). The lungs are the most common site for osteosarcoma metastasis, although it can also spread to other bones or organs via the bloodstream or lymphatic system (13). Early detection of metastasis is critical for improving the chances of successful management in osteosarcoma patients.

The tumor microenvironment (TME) has gained significant attention because it plays a crucial role in tumor progression, metastasis, and treatment outcomes (14, 15). The TME refers to the surrounding microenvironment in which tumors exist, including both cellular and noncellular components such as noncancerous cells, blood vessels, and extracellular matrix (ECM) components. Noncancerous cells include immune cells, fibroblasts, and other cell types. Blood vessel formation supplies oxygen and nutrients to tumor cells and provides a pathway for tumor metastasis (16). The TME of osteosarcoma includes osteoblasts, osteocytes, osteoclasts, pericytes, endothelial cells, fibroblasts, mesenchymal stem cells, and various immune cells, such as lymphoid and myeloid cells, as well as the ECM (17, 18).

A better understanding of the TME in osteosarcoma, including the interactions between cancer cells and noncancerous cells and the mechanisms of metastasis, is crucial for developing effective tumor treatments (19). Tumor cells interact with their surrounding TME in complex ways, influencing their biological behavior and response to therapies. Immunotherapy often targets the TME to enhance the immune response against cancer by focusing on specific components within the TME. Immunotherapy includes treatments such as checkpoint inhibitors, cytokines, cancer vaccines, and chimeric antigen receptor (CAR) T-cell therapy (20). CAR T-cell therapy has demonstrated promising results in combating osteosarcoma (21). Targeting the TME has opened new avenues for cancer treatment. In this study, we used single-cell RNA sequencing (scRNA-seq) to reveal the communications between tumor microenvironment components and tumor metastasis in osteosarcoma.

## 2 Materials and methods

### 2.1 Data collection

The GSE152048 dataset from the GEO (https://www.ncbi.nlm.nih.gov/geo/query/acc.cgi?acc=GSE152048) database was downloaded. The scRNA-seq data were obtained from the GSE152048 dataset. We selected two primary osteosarcoma samples (BC2, BC22) and two metastasis osteosarcoma samples (BC10, BC17) for further analysis. Additionally, the GSE14359 and GSE49003 datasets were downloaded from the GEO database. The GSE14359 dataset includes 10 primary osteosarcoma samples and 8 metastatic osteosarcoma samples, while the GSE49003 dataset includes 6 primary osteosarcoma samples and 6 metastatic osteosarcoma samples.

### 2.2 Differential and functional analysis

Differentially expressed genes (DEGs) were explored in osteosarcoma cases with primary and metastatic features using R software and the *limma* package. A cutoff of |log2 (fold change)| > 0.5 and *p* < 0.05 was used to determine the DEGs. Metastasis-related genes were dissected when DEGs were shared between the GSE14359 and GSE49003 datasets. Gene ontology (GO) and Kyoto Encyclopedia of Genes and Genomes (KEGG) pathway enrichment analyses were performed to examine metastasis-related genes. Several R packages, including “clusterProfiler”, “org.Hs.eg.db”, and “enrichplot” were used for functional analysis. A *p*-value < 0.05 was considered a significant enrichment.

### 2.3 Protein–protein interaction network construction

A protein–protein interaction network was established using the STRING database to analyze the metastasis-associated genes. The network was reconstructed and visualized using Cytoscape software. The *cytohubba* plugin was employed to identify hub genes.

### 2.4 Tumor microenvironment analysis

Tumor purity was calculated for each tumor sample. An estimate algorithm was used to detect the scores for immune cells and stromal cells (22). The estimate R package (https://bioinformatics.mdanderson.org/estimate/rpackage.html) was utilized to determine the stromal score, immune score, ESTIMATE score, and tumor purity of different clusters in osteosarcoma patients from the GSE14359 and GSE49003 datasets.

### 2.5 Tumor immune infiltration analysis

The abundance of different cell types was explored and estimated in a mixed-cell population using the CIBERSORT approach, developed by the Newman group (23). The CIBERSORT approach was applied to estimate the differences in 22 immune cell types among osteosarcoma patients in the GSE14359 and GSE49003 datasets. This analysis was performed using the R package “e1071” (Version: 1.7-3) as a precondition.

### 2.6 scRNA-seq data processing and analysis

The scRNA-seq data were further analyzed using the Seurat package (version 3.1.5; http://satijalab.org/seurat/) in R software (version 3.6.1) for each sample (24). In each sample, the Seurat object containing gene expression data was represented using the Read10× () function. Three quality control criteria were applied to exclude low-quality cells: (1) genes observed in fewer than 10 cells; (2) cells with fewer than 200 total observed genes; and (3) cells with more than 5% of genes expressed in mitochondria.

Gene expression data were normalized by converting values to the natural logarithm after multiplying the gene fraction by 10,000. The top 2,000 highly variable genes were selected, scaled, and analyzed using principal component analysis (PCA). Significant principal component (PC) values for cell clustering were determined using Dimheatmap and JackStrawPlot. Subsequently, t-distributed stochastic neighbor embedding (t-SNE) was performed to identify cell classification, and the maker genes of each cluster were screened by the FindAllMarkers function, with cutoff values of |log2 fold change(FC)| > 1, the cell population ratio  > 0.25, and an adjusted *p*-value < 0.05. The SingleR (version: 1.0.6) package [16] was used for cell cluster annotation, drawing on data from the celldex package HumanPrimaryCellAtlasData. The monocle (version: 2.14.0) package (25) was used to reconstruct cell differentiation trajectories. Dimension reduction analysis was conducted using the reduceDimension function with reduction_method = “DDRTree” and max_components = 2. Characteristic genes of different cell states were filtered using the criteria of |log2FC| > 1 and adjusted *p*-value < 0.05 for downstream analysis.

### 2.7 Cell communication analysis using CellChat

Intercellular communications were clarified through ligand–receptor interactions and analyzed using the R package CellChat (26). Firstly, all ligand–receptor pairs were required to be expressed in the 8 cell subpopulations, and a ligand–receptor subnetwork was developed and saved in the human ligand–receptor pair database CellChatDB. Secondly, a biologically significant cell–cell communication network was constructed, mediated by ligand–receptor interactions. Thirdly, interacting ligand–receptor pairs related to the TNF, ANGPTL, CXCL, and IL families were further explored to examine the relationships among cell types.

## 3 Results

### 3.1 Identification of metastasis-associated genes

**Figure 1:**
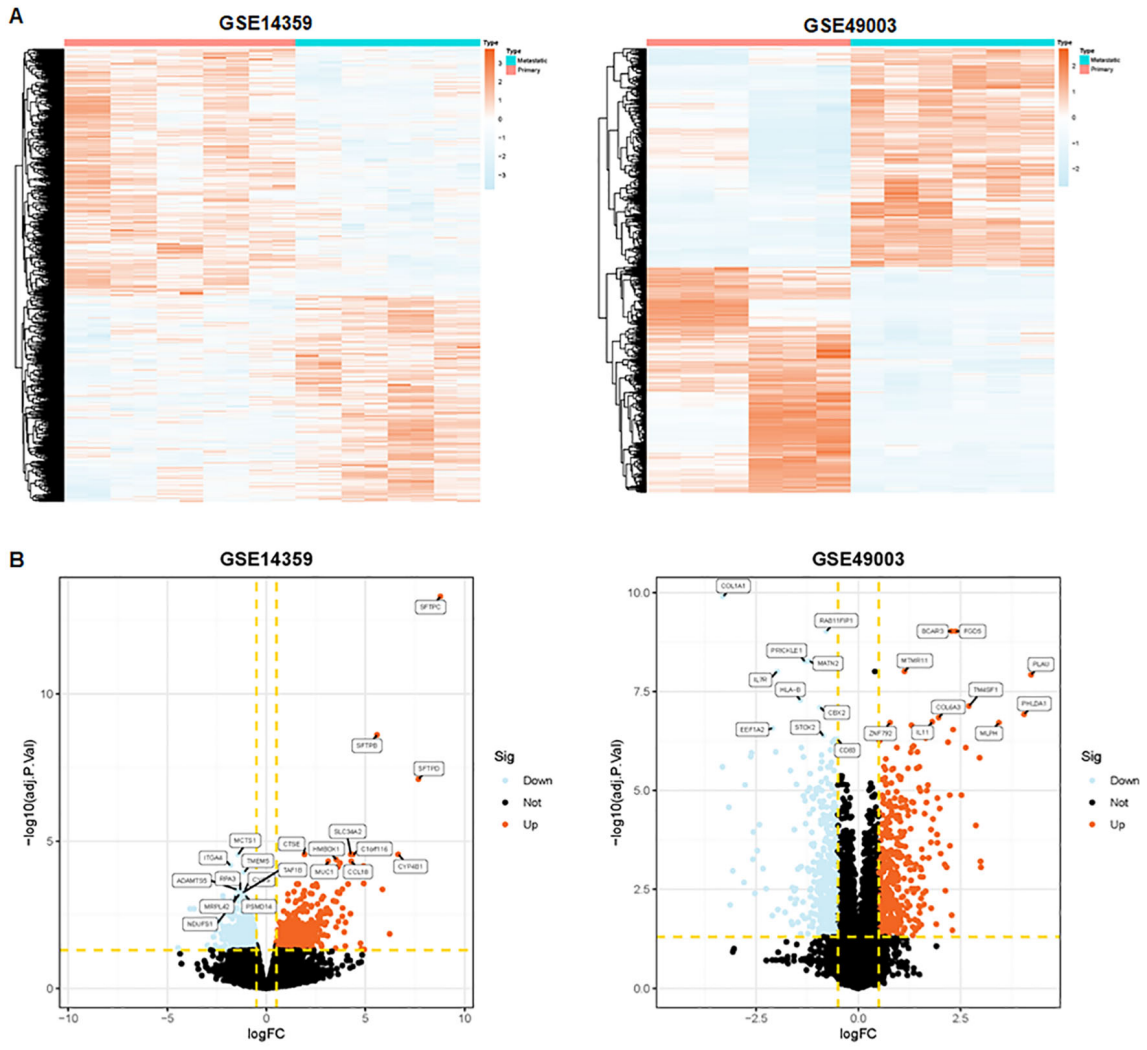
Identification of metastasis-related genes. **(A)** Heatmaps depicting metastasis-related genes in the GSE14359 and GSE49003 datasets. **(B)** Volcano plot illustrating differentially expressed genes in primary and metastatic osteosarcoma tissues.

**Figure 2:**
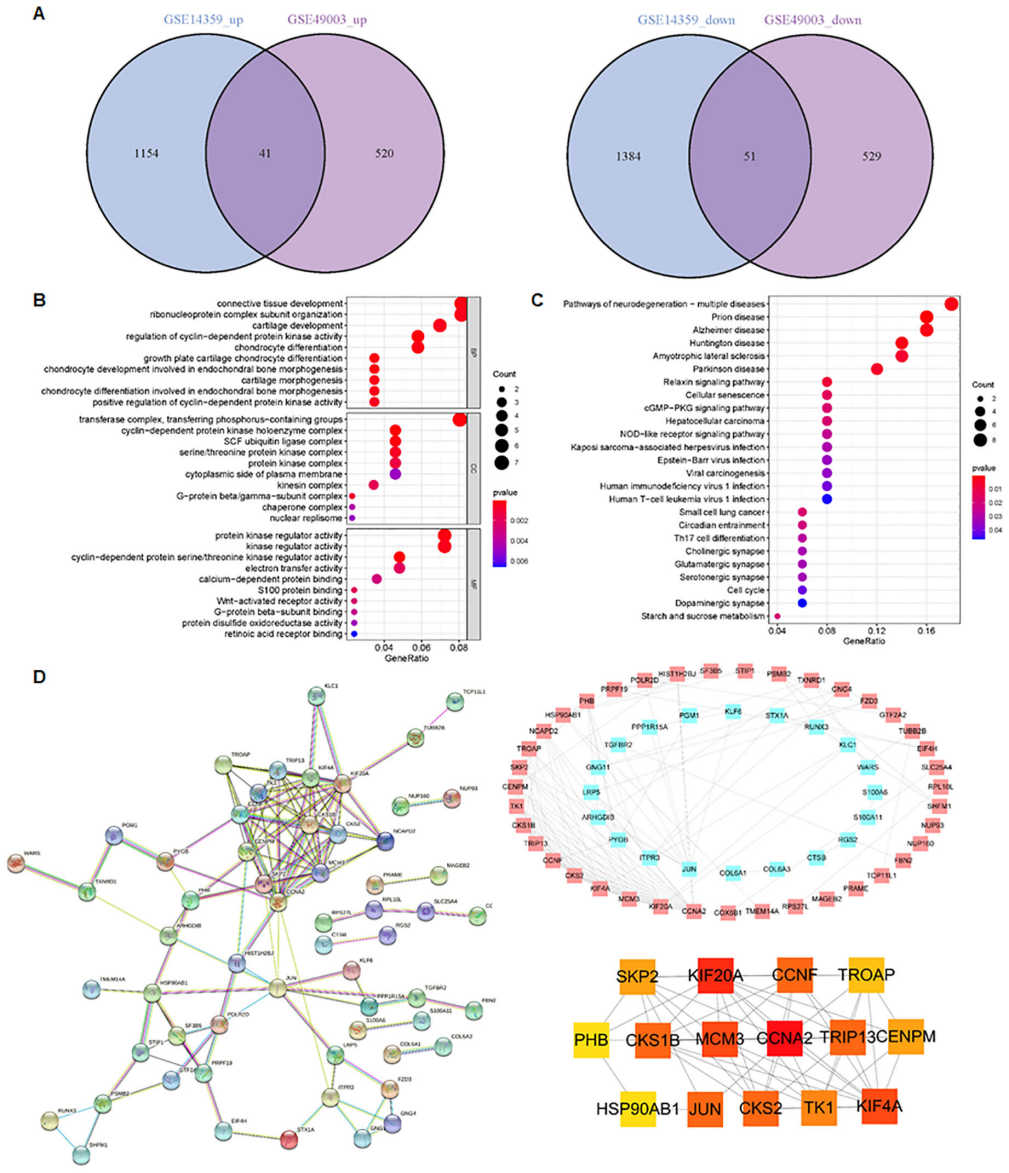
Identification of metastasis-related pathways. **(A)** In total, 41 DEGs were upregulated and 51 DEGs were downregulated in both the GSE14359 and GSE49003 datasets. **(B)** GO enrichment analysis of the 92 DEGs. **(C)** KEGG pathway analysis of the 92 DEGs. **(D)** PPI network of the DEGs generated using the STRING database.

To identify metastasis-associated genes in osteosarcoma, we selected two osteosarcoma datasets in the GEO database: GSE14359 and GSE49003. These datasets include numerous primary and metastatic osteosarcoma tissues. In the GSE14359 dataset, 1,195 genes were upregulated and 1,435 genes were downregulated, while in the GSE49003 dataset, 561 genes were upregulated and 580 genes were downregulated (**Figures 1A, B**). Among these, 92 DEGs were shared between both datasets, with 41 DEGs upregulated and 51 DEGs downregulated (**Figure 2A**).

### 3.2 Identification of metastasis-associated pathways

GO and KECG enrichment analyses were performed on the 92 DEGs (**Figures 2B, C**). The GO analysis revealed that the regulation of cyclin-dependent protein kinase activity, growth plate cartilage chondrocyte differentiation, chondrocyte development involved in endochondral bone morphogenesis, and chondrocyte differentiation were enriched in the 92 DEGs (**Figure 2B**). In terms of cellular components, the cyclin-dependent protein kinase holoenzyme complex, transferase complex, transferring phosphorus-containing groups, SCF ubiquitin ligase complex, serine/threonine protein kinase complex, and G-protein beta/gamma-subunit complex were found to be enriched (**Figure 2B**). Regarding molecular function, cyclin-dependent protein serine/threonine kinase regulator activity, protein kinase regulator activity, kinase regulator activity, electron transfer activity, and S100 protein binding were involved (**Figure 2B**).

### 3.3 KEGG analysis

**Figure 3:**
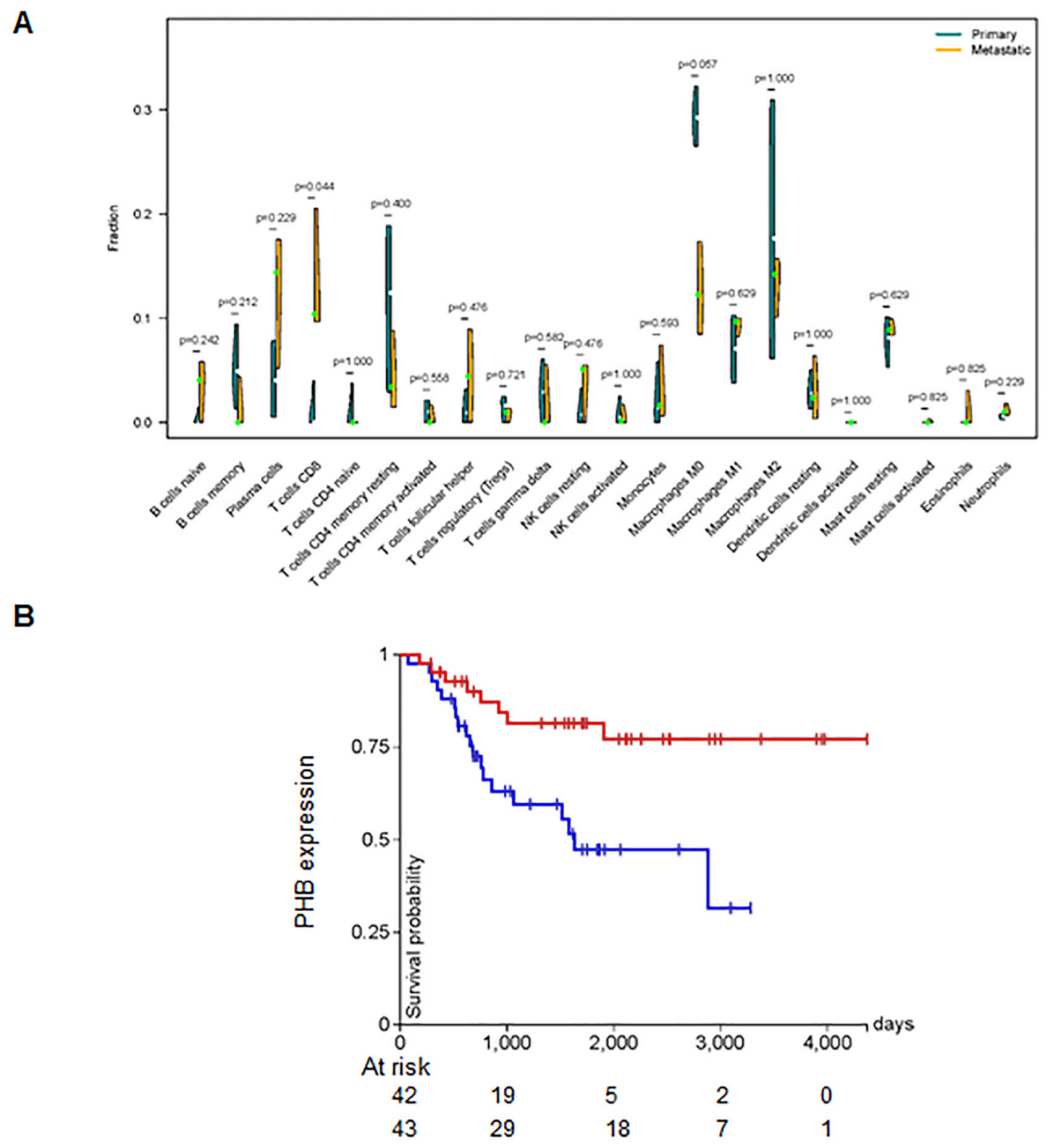
CD8+ T cells are associated with metastasis. **(A)** Difference in the fraction of 22 immune cells across different tumor types. The proportion of CD8+ T cells was higher in metastatic tissues. **(B)** Kaplan–Meier survival analysis showing that high expression of PHB is associated with a favorable prognosis in osteosarcoma patients.

KEGG pathway analysis showed that the 92 DEGs were involved in regulating the cGMP-PKG and NOD-like receptor signaling pathways (**Figure 2C**). Furthermore, the protein–protein interaction (PPI) network of the DEGs was generated using the STRING database (**Figure 2D**). We found the top 15 hub genes by cytoHubba plug-in of the Cytoscape software, including Skp2, KIF20A, CCNF, TROAP, PHB, CKS1B, MCM3, CCNA2, TRIP13, CENPM, Hsp90AB1, JUN, CKS2, TK1, and KIF4A (**Figure 2D**). Among these, CCNA2 and KIF20A emerged as the top two candidates within the PPI network (**Figure 2D**). Additionally, we observed that the proportion of CD8+ T cells was higher in metastatic tissues compared to primary osteosarcoma tissues (**Figure 3A**). Notably, high expression of PHB was associated with a favorable prognosis in osteosarcoma patients, as shown by Kaplan–Meier survival analysis (**Figure 3B**).

### 3.4 scRNA-seq data analysis

**Figure 4:**
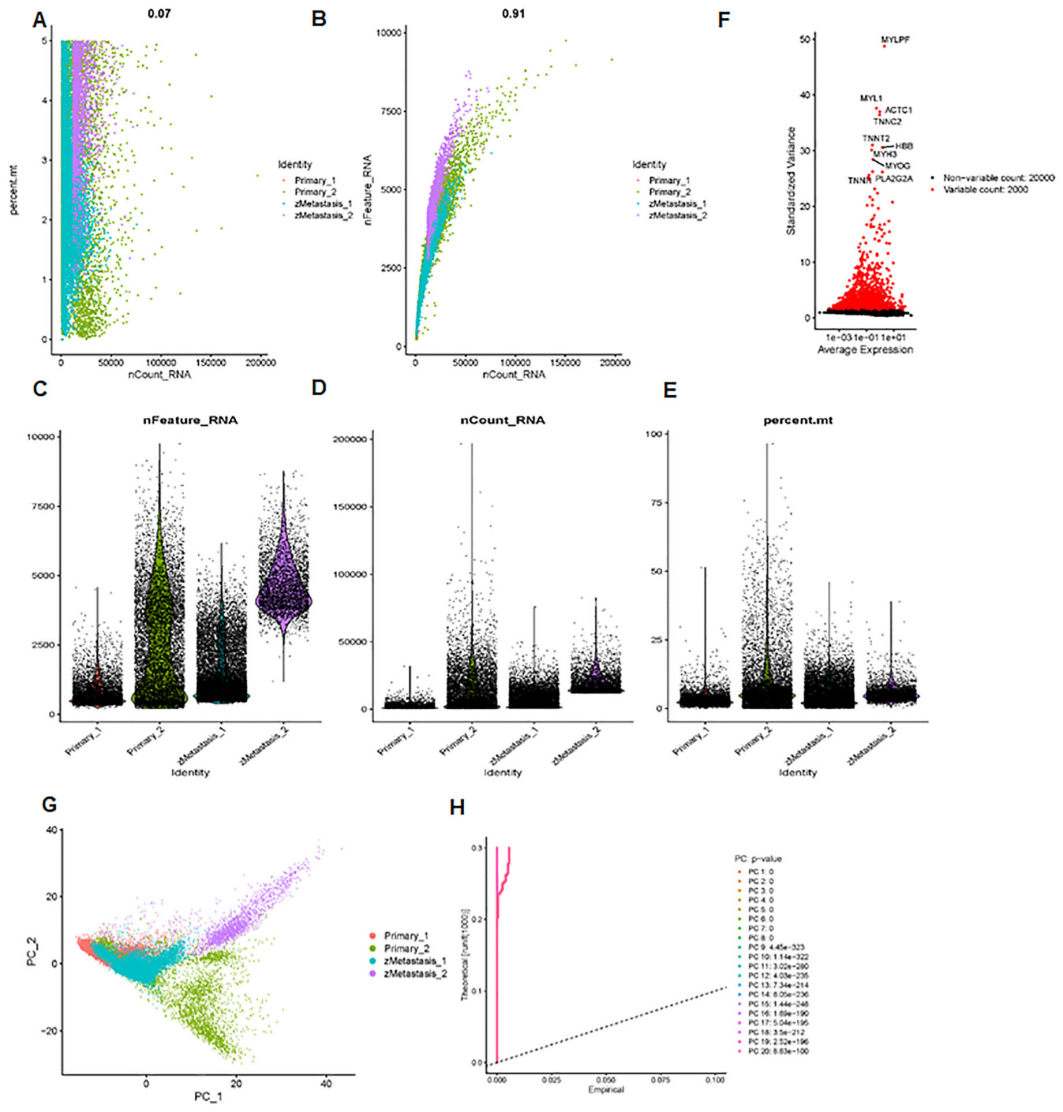
scRNA-seq data analysis. **(A)** Pearson correlation between sequencing depth and mitochondrial gene expression. **(B)** The sequencing depth has a positive correlation with the number of genes, with a coefficient of 0.91. **(C–E)** The number of genes and sequencing depth across 24,375 cells are shown. **(F)** Volcano plot data show highly variable genes across cells, including MYL1, ACTC1, MYLPF, TNNC2, TNNT2, MYH3, MYOG, and PLA2G2A. **(G)** Principal component analysis was performed based on these highly variable genes. **(H)** Determination of principal component value using the JackStraw function.

**Figure 5:**
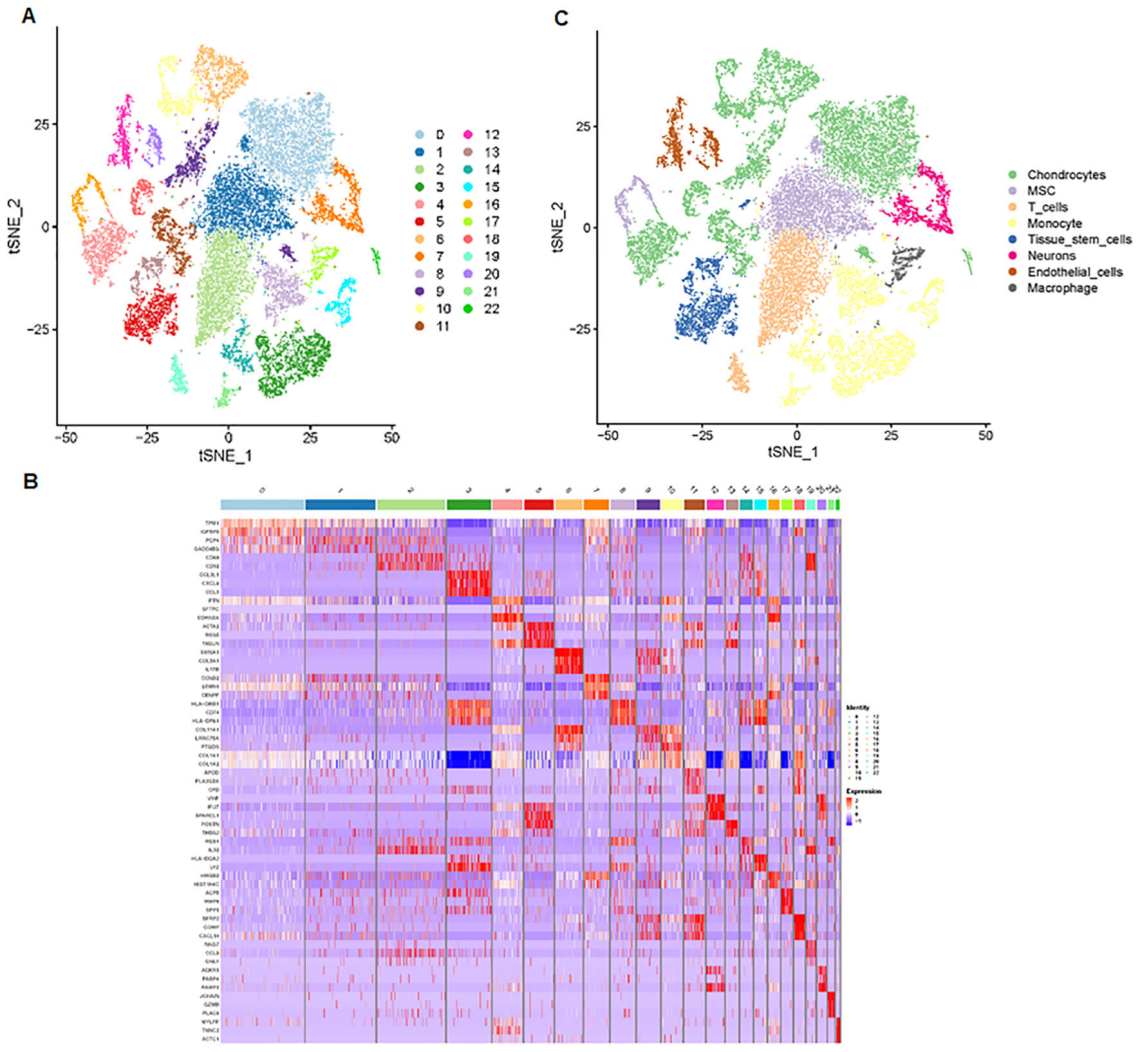
Cell clusters and cell types are identified. **(A)** The t-SNE algorithm was applied to reduce the nonlinear dimension, identifying 23 clusters within the single-cell samples. **(B)** Heatmap data showing the top three marker genes for each cluster. **(C)** The 23 cell clusters were classified into eight cell types, including chondrocytes, MSC, T cells, monocytes, tissue stem cells, neurons, endothelial cells, and macrophages.

Next, we analyzed a total of 24,375 single-cell samples. First, we used Pearson’s correlation to measure the relationship between sequencing depth and mitochondrial gene expression (**Figure 4A**). Sequencing depth had a positive correlation with the number of genes, with a coefficient of 0.91 (**Figure 4B**). We also demonstrated the number of genes and sequencing depth in 24,375 cells from four osteosarcoma patients (**Figures 4C–E**). A volcano plot highlighted the highly variable genes across the cells, including MYL1, ACTC1, MYLPF, TNNC2, TNNT2, MYH3, MYOG, and PLA2G2A (**Figure 4F**). The principal component analysis (PCA) was performed based on these highly variable genes (**Figure 4G**). The determination of significant components was guided by the JackStraw function, with 20 principal components selected (**Figure 4H**). We then applied the t-SNE algorithm for nonlinear dimension reduction, ultimately identifying 23 clusters within the single-cell samples (**Figure 5A**). These 23 cell clusters were marked by a total of 4,164 genes, with the top three marker genes of each cluster shown in a heatmap (**Figure 5B**). Furthermore, the 23 cell clusters were classified into eight cell types: chondrocytes, MSC, T cells, monocytes, tissue stem cells, neurons, endothelial cells, and macrophages (**Figure 5C**).

### 3.5 Analysis of top 15 hub genes

**Figure 6:**
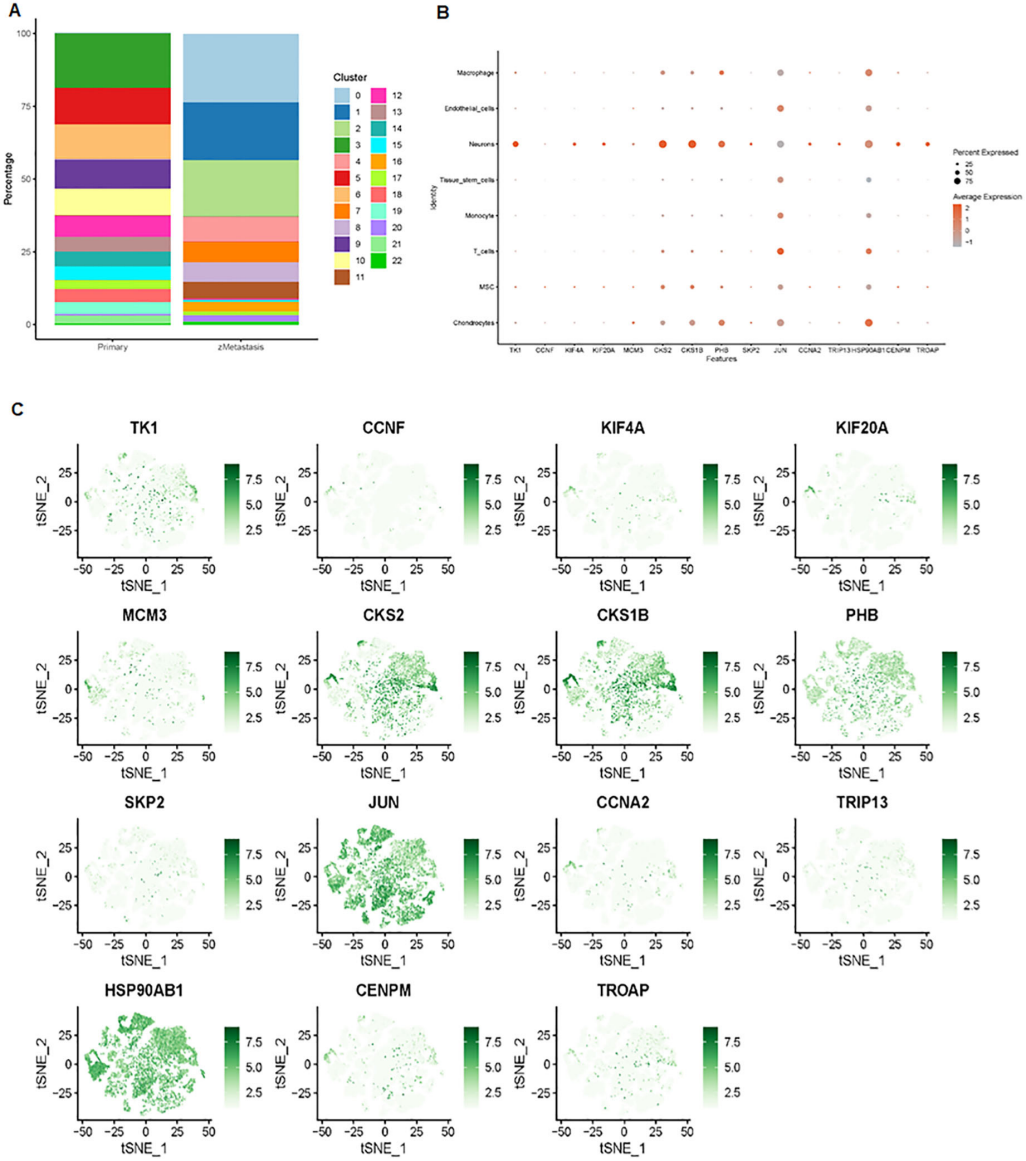
Expression analysis of the top 15 hub genes. **(A)** Distribution of 23 clusters in primary and metastatic tissues. **(B)** Bubble plot showing the expression levels of the top 15 hub genes across the 23 cell clusters. **(C)** tSNE maps illustrating the expression of the top 15 genes within the 23 cell clusters.

**Figure 7:**
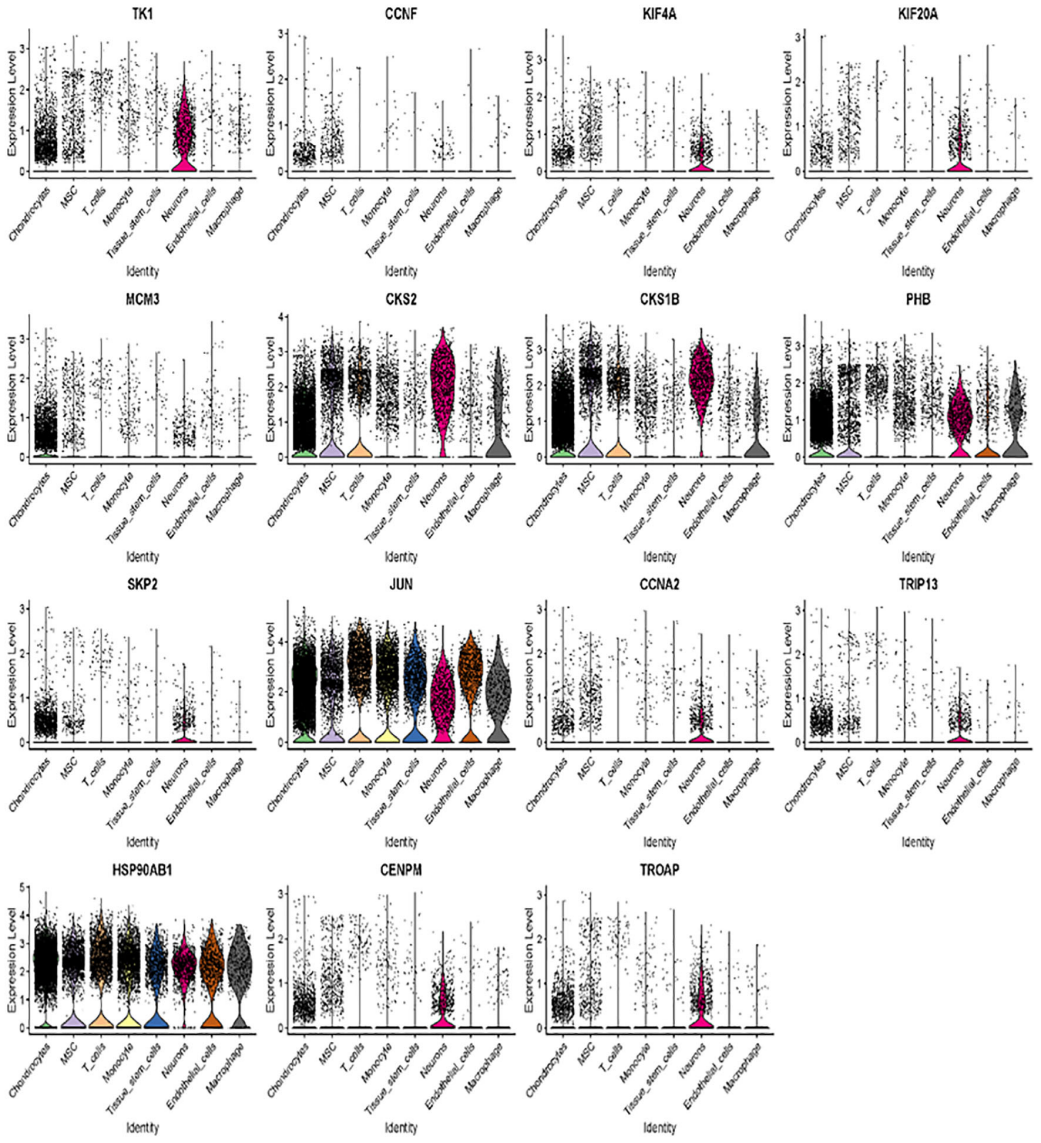
Across various cell types, 15 hub genes are expressed.

We further examined the expression and function of the top 15 hub genes across the eight cell types in osteosarcoma (**Figures 6A, B**). We found that TK1, CCNF, KIF4A, and KIF20A had higher expression levels in neurons, MSC, and chondrocytes (**Figure 6B**). Skp2, CCNA2, TRIP13, CENPM, and TROAP were highly expressed in neurons. JUN was more highly expressed in T cells and endothelial cells, while its expression was lower in neurons. Hsp90AB1 showed higher expression in chondrocytes and T cells but lower expression levels in tissue stem cells, monocytes, and MSC (**Figures 6B, C**). These 15 hub genes were expressed across various cell types (**Figure 7**), suggesting that they play different roles in osteosarcoma development (**Figure 7**).

### 3.6 Cell–cell communications

**Figure 8:**
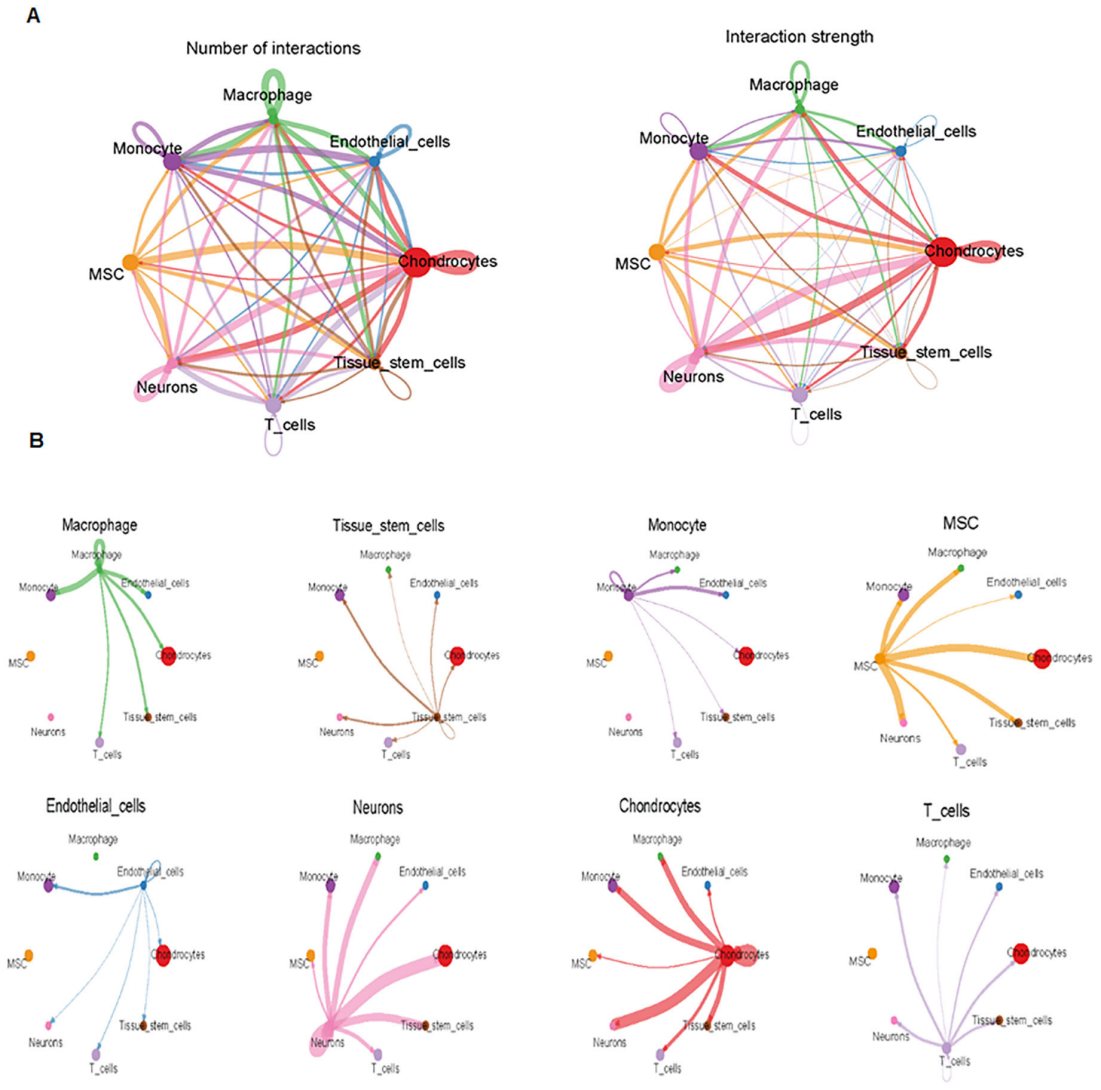
Comparison of cell–cell interactions among various cell types. **(A)** CellChat analysis of cell–cell communication across various cell types, showing an overview of the different interactions in osteosarcoma. Interaction number (left side) and interaction strength (right side) are illustrated. **(B)** Cell–cell communication analysis across eight cell types.

**Figure 9:**
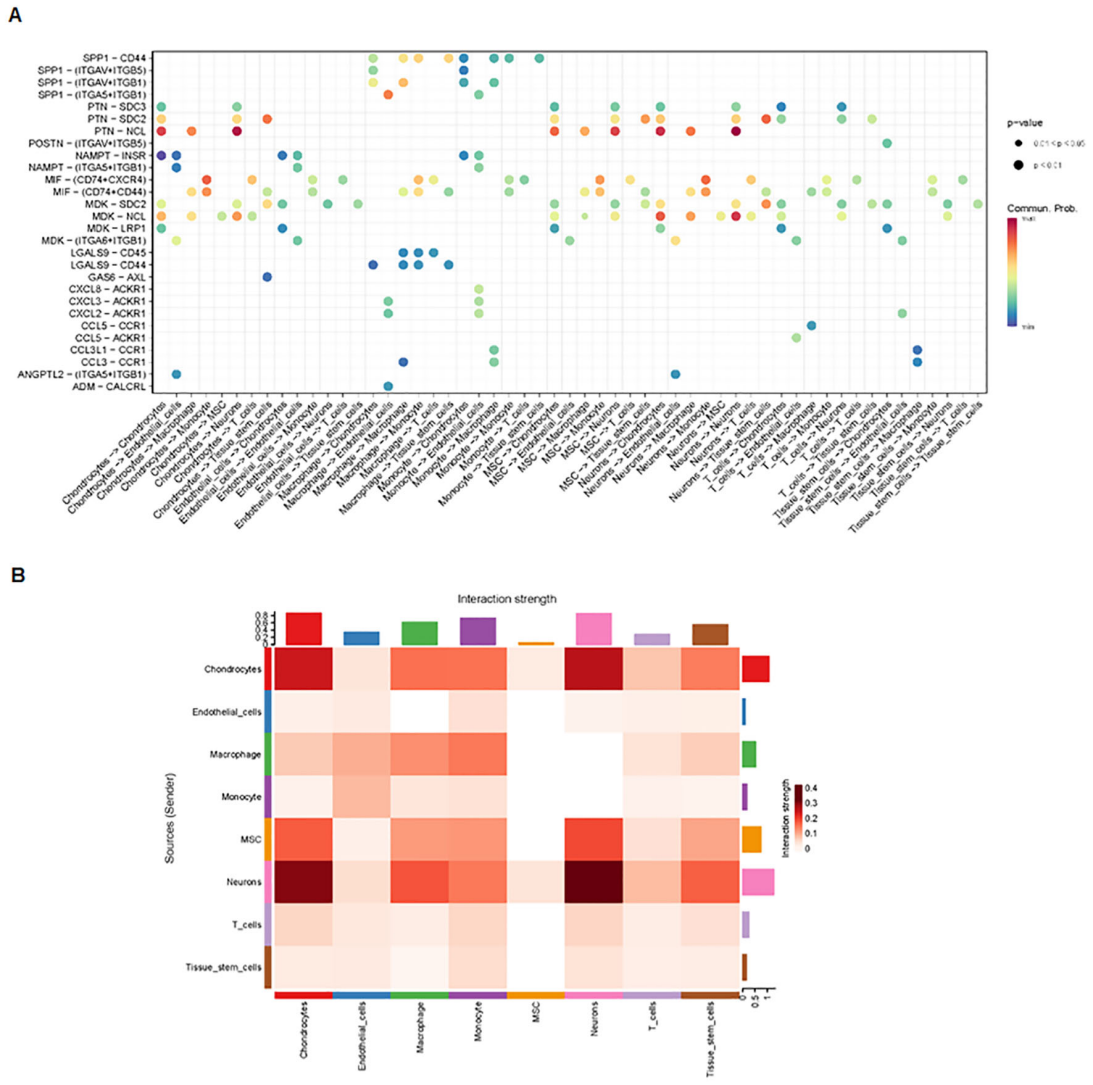
Interactions between various cell groups. **(A)** NetVisual bubble plot showing interactions between specific cell groups. **(B)** Heatmaps depicting interactions among eight cell types.

**Figure 10:**
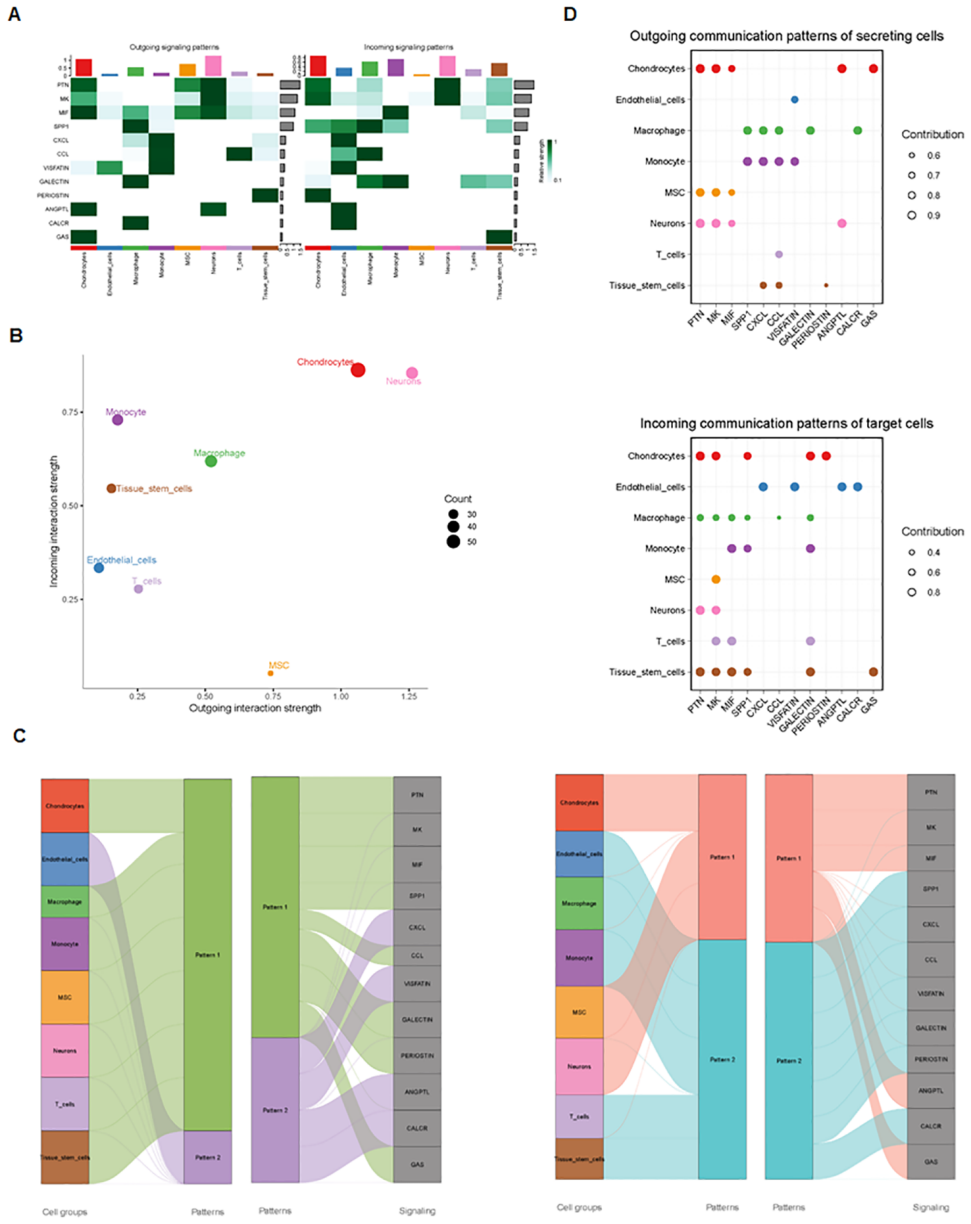
Outgoing and incoming signaling in each cell population. **(A)** Comparison of outgoing and incoming signaling associated with each cell population. **(B)** Signaling dots are illustrated. **(C)** Incoming and outgoing communication patterns across various cell types. **(D)** Contribution of cell populations to signaling pathways, with outgoing communication patterns of secreting cells (top) and incoming communication patterns of target cells (bottom) illustrated.

Finally, we analyzed cell–cell communication between different cell types using the CellChat tool. We found that there was an interaction between different cell types (**Figure 8A**). Each cell type interacted differently with other cell types (**Figure 8B**). Using netVisual bubble plots, we observed significant interactions between certain cell groups and others (**Figures 9A, B**). We also compared the outgoing or incoming signaling associated with each cell population (**Figures 10A, B**). The incoming communication of target cells and outgoing communication patterns of secreting cells highlighted the correspondence between cell groups and signaling pathways (**Figure 10C**). Additionally, we discovered the contribution of different cell populations to all signaling pathways (**Figure 10D**).

## 4 Discussion

The TME plays a crucial role in the progression and metastasis of osteosarcoma (27). Comprehensive bioinformatics analyses have been utilized to explore the mechanisms underlying osteosarcoma development, prognosis, immune microenvironment, and drug sensitivity. For example, one bioinformatics study identified the oncogenic function of FoxM1 and its association with TME, prognosis, and drug resistance in osteosarcoma (28). Another study identified 28 metastasis-related genes in osteosarcoma, including 16 upregulated and 12 downregulated genes. GO analysis suggested that these genes were enriched in Wnt and kinase pathways, bone morphogenesis, and development. KEGG pathway analysis showed that these genes were involved in the Wnt, JAK-STAT pathway, and cytokine–cytotoxic receptor interactions and were also associated with immunity in osteosarcoma patients (29). In this study, we identified 41 upregulated and 51 downregulated DEGs in both the GSE14359 and GSE49003 databases.

ScRNA-seq is a powerful tool for evaluating intratumoral heterogeneity in various cancers, including osteosarcoma (30–32). For instance, the ScRNA transcriptome has revealed the heterogeneity and immunosuppressive roles of regulatory T cells (Tregs) in osteosarcoma, with patients displaying high Tregs risk showing sensitivity to axitinib, sunitinib, and sorafenib. This finding suggests that Treg heterogeneity could open a new window for osteosarcoma treatment (33). Li et al. used ScRNA-seq data to identify natural killer (NK) cell marker genes, finding that low-risk osteosarcoma patients had high levels of infiltrating immune cells, particularly naïve CD4 and CD8 T cells, in an immunosuppressive microenvironment (34). Yi et al. reported that a proangiogenic tumor-associated macrophages (TAMs) gene risk signature could serve as a prognostic biomarker and therapeutic target in osteosarcoma (35). He et al. analyzed scRNA-seq data from lung metastasis samples and identified intratumoral heterogeneity in osteosarcoma lung metastasis, highlighting the abundance of T cells, particularly CD8+ T cells, which showed low immune checkpoints (36). In our study, we found that CD8+ T-cell proportions were higher in metastatic tissues than in primary osteosarcoma tissues.

RNA-sequencing of individual cells from lung metastatic tissues, primary tissues, and recurrent osteosarcoma tissues revealed 11 cell clusters in osteosarcoma, with lower osteoclast infiltration in chondroblastic, recurrent, and lung metastatic tissues, suggesting intratumoral heterogeneity and an immunosuppressive microenvironment in advanced osteosarcoma (37). Another study using single-cell transcriptomics observed the complexity of the TME in osteosarcoma patients without chemotherapy, identifying nine major cell types and highlighting T-cell depletion as a key feature (38). Moreover, one study integrating scRNA-seq, microarray, and bulk RNA-seq data identified tumor stem cell-related genes associated with survival, TME, and immune infiltration status in osteosarcoma, such as CKLF, DKK1, and Myc (39). Based on the scRNA-seq data, 11 clustered cell types were identified, and cell–cell interactions were found among various cell types in recurrent, metastatic, and primary osteosarcomas. NF-kB pathway was critically involved in controlling the TME in osteosarcoma (40). Herein, we identified eight cell types, including chondrocytes, MSC, T cells, monocytes, tissue stem cells, neurons, endothelial cells, and macrophages in osteosarcoma using scRNA-seq.

Based on scRNA-seq and bulk RNA-seq, Qin et al. revealed ATG16L1 as a potential immune signature and prognostic factor in osteosarcoma (41). Similarly, several survival-related genes, including MUC1, COL13A1, KAZALD1, and JAG2, were identified as prognostic markers in osteosarcoma by scRNA-seq and TARGET analysis (42). Osteoclast differentiation-related genes such as LOXL1, SERPINE2, FAM207A, TPM1, ST3GAL4, and S100A13 were also linked to prognosis (43). Additionally, genes like COL6A1, COL6A3, and MIF were associated with lung metastasis, indicating that a highly invasive subcluster in osteosarcoma correlated with a poor prognosis (44). Our study reported that high expression of PHB was associated with a favorable prognosis in osteosarcoma patients.

Using scRNA-seq, a chemoresistant risk-scoring model was established, demonstrating that scRNA-seq could be a valuable tool for evaluating chemoresistance in osteosarcoma (45). Genes such as EGFL7 and VEGF were differentially expressed in osteosarcoma (46), and NR4A1 was found to play a key role in osteosarcoma pathophysiology (47). Single-cell transcriptomics have also been employed to explore the functions of cancer-associated fibroblasts in the TME of recurrent osteosarcoma (48). A five-gene panel, including BAMBI, TMCC2, NOX4, DKK1, and CBS, was revealed as a prognostic model via scRNA-seq and bulk RNA-seq analysis (49). Similar studies integrating bulk RNA-seq and scRNA-seq have been conducted to explore the TME and metabolic pattern in osteosarcoma (50). Moreover, scRNA-seq has been used to characterize TME phenotypes in pleural effusion and tumor tissues from advanced-stage osteosarcoma patients (51). Seven genes, including ANXA1, FKBP11, SP7, TPM1, FDPS, IFITM5, and SQLE, were identified as prognostic biomarkers in mesenchymal stem cells using scRNA-seq (52). LPAR5 was identified as a potential indicator for TME remodeling and a therapeutic target for osteosarcoma based on scRNA-seq data (53). Additionally, five key genes, including CD4, RUNX2, OMD, COL9A3, and JUN, were found to be associated with osteosarcoma progression (54). GNG4 (55) and C1Q+ tumor-associated macrophages (56) were reported to predict osteosarcoma prognosis using scRNA-seq. Our study identified 15 hub genes expressed across various cell types, including TK1, CCNF, KIF4A, KIF20A, Skp2, CCNA2, TRIP13, CENPM, and TROAP.

Among these hub genes, Skp2 has been reported as an E3 ubiquitin ligase involved in osteosarcoma progression. Studies have shown that suppression of Skp2 attenuates cell proliferation and invasion in osteosarcoma, while overexpression of Skp2 enhances cell growth and motility (57, 58). Additionally, Skp2 has been implicated in promoting epithelial-mesenchymal transition (EMT) in osteosarcoma cells (59) and facilitating stemness and tumor progression through interaction with p27 (60). Targeting Skp2 inhibition has demonstrated anticancer effects, leading to survival benefits in osteosarcoma (61). It has been known that retinoblastoma (RB) and p53 pathways are critically involved in aging, senescence, and tumorigenesis (62, 63). Skp2 knockout has been shown to trigger immune infiltration and improve prognosis in Rb1/p53-deficient mice with osteosarcoma (64).

Recently, Lu et al. used integrative analysis of single-cell and bulk transcriptome data to discover neutrophil-related genes in osteosarcoma, such as C3AR1 and FCER1G (65). Zheng et al. employed single-cell transcriptomics to uncover chemotherapy-mediated remodeling of the TME in osteosarcoma (66). Yi et al. identified a pro-protein synthesis osteosarcoma subtype using scRNA-se1, which could be useful for predicting prognosis and treatment (67). Additionally, Truong et al. discovered a single-cell differentiation landscape in osteosarcoma, which may aid in developing targeted therapy (68).

## 5 Conclusion

In this study, we identified metastasis-associated genes and pathways in osteosarcoma. Additionally, we verified the presence of 8 cell types, including chondrocytes, MSC, T cells, monocytes, tissue stem cells, neurons, endothelial cells, and macrophages, in osteosarcoma using scRNA-seq. Furthermore, 15 hub genes were observed in various cell types. Our study provides valuable insights into the metastatic mechanisms of osteosarcoma. However, it is important to acknowledge the limitations of this study. The findings from scRNA-seq have not been validated through cellular experiments and mouse studies. Additionally, metastasis-associated genes and pathways in osteosarcoma have not been clarified through clinical studies. To validate our findings, *in vitro* experiments and *in vivo* studies are required. It is also necessary to examine the expression of the 15 hub genes in metastatic tissues of osteosarcoma patients. By unraveling the complex interactions between osteosarcoma cells and the surrounding microenvironment at the single-cell level, we can identify novel therapeutic targets to inhibit metastasis and improve treatment outcomes. Moreover, scRNA-seq could be instrumental in uncovering mechanisms of resistance to current therapies, leading to the development of combination therapies that overcome resistance. Future research should explore how the TME evolves during tumor progression and how certain microenvironmental niches contribute to osteosarcoma metastasis.

## Data Availability

The original contributions presented in the study are included in the article/supplementary material. Further inquiries can be directed to the corresponding authors.
